# Development and validation of a machine learning model for predicting occupational stress among primary and secondary school teachers

**DOI:** 10.3389/fpubh.2026.1767113

**Published:** 2026-04-08

**Authors:** Jinyu Wang, Xinran Zhang, Sheng Li

**Affiliations:** 1 Department of Occupational and Environmental Health, School of Public Health, Lanzhou University, Lanzhou, China; 2 The Second Hospital and Clinical Medical School, Lanzhou University, Lanzhou, China; 3 Department of Public Health, Lanzhou Second People’s Hospital, Lanzhou, China

**Keywords:** machine learning, occupational stress, predictive models, primary and secondary school teachers, XGBoost, SHAP

## Abstract

**Background:**

To address the limitations of traditional linear tools in predicting teacher occupational stress, this study aimed to develop and validate machine learning models using easily obtainable, self-reported data.

**Method:**

A cross-sectional study of 2,832 in-service teachers in Lanzhou, China, was conducted. The presence of occupational stress, defined by the Core Occupational Stress Scale, was modeled using sociodemographic, work-related, and lifestyle factors. The dataset was partitioned into training (80%) and validation (20%) sets to compare six machine learning models: Extreme Gradient Boosting (XGBoost), Light Gradient Boosting Machine (LightGBM), a Backpropagation Neural Network, Elastic Net, Logistic Regression, and a Support Vector Machine. Model performance was evaluated using the Area Under the Receiver Operating Characteristic Curve, accuracy, F1-score, and Decision Curve Analysis. The optimal model was interpreted using the SHapley Additive exPlanations method.

**Results:**

The prevalence of occupational stress was 33.3%. On the validation set, the Extreme Gradient Boosting model demonstrated the best performance, with an Area Under the Curve of 0.620, an accuracy of 0.603, and an F1-score of 0.682. Decision Curve Analysis confirmed this model provided the highest net benefit. The LightGBM and Neural Network models exhibited significant overfitting. SHapley Additive exPlanations analysis identified weekly exercise time, sex, and age as the most influential predictors. A user-friendly, web-based tool was developed from the final model.

**Conclusion:**

Machine learning, particularly the Extreme Gradient Boosting algorithm, can effectively predict occupational stress in teachers. This approach offers a promising tool for early identification, enabling targeted interventions.

## 1 Introduction

Occupational stress among teachers has escalated into a critical global public health challenge, with detrimental effects that not only erode the mental and physical well-being of individuals but also profoundly threaten the stability of educational systems and the quality of development for the next generation (1). Amidst deepening educational reforms, primary and secondary school teachers, as frontline implementers, are subjected to immense pressure from a confluence of factors including excessive workloads, societal expectations, and complex interpersonal dynamics, rendering them a particularly vulnerable population (2). The pathways linking chronic stress to deteriorating teacher health are both complex and alarming: prolonged overwork and time pressure can trigger chronic fatigue and cognitive decline (3); the emotional labor required to manage student behavior and communicate with parents is a core driver of burnout and emotional exhaustion (4); and the technostress accompanying the digital transformation of education further exacerbates this high-demand professional environment.

However, current strategies for risk assessment and intervention in teacher occupational stress suffer from a significant lag. Traditional assessment tools, such as self-report scales, while valuable, are constrained by static and linear analytical frameworks. These frameworks are often insufficient to capture the complex, non-linear interactions among the multifaceted risk factors underlying occupational stress (5). This limitation not only impedes the opportunistic and large-scale early screening of high-risk teachers but also perpetuates a concerning reality: a large, vulnerable teacher population lacks an accessible and reliable tool for proactive risk stratification.

To overcome the bottlenecks of conventional methods and address this gap in preventative health strategy, this study introduces an advanced machine learning (ML) paradigm (6). We aim to develop and validate an intelligent predictive model using a representative sample of primary and secondary school teachers in Lanzhou. This model will be based exclusively on non-invasive and easily obtainable variables (7). Unlike traditional statistical models, machine learning algorithms can autonomously learn complex non-linear patterns and feature interactions from high-dimensional data, thereby offering superior predictive accuracy and individualized insights (8).

The core objective of this research is to construct a model that is not only highly accurate but also interpretable (9). By integrating teachers’ social demographic characteristics, work-related factors, and lifestyle variables, we seek to develop a reliable and efficient online self-assessment tool. This tool is designed to empower teachers to engage in proactive health risk management and to facilitate the early identification and warning of occupational stress (10). Ultimately, we envision this data-driven solution providing an evidence-based foundation for educational administrators to implement precise and personalized interventions. The goal is to improve the health trajectory and well-being of this crucial professional group, fostering a more resilient educational ecosystem.

## 2 Methods

### 2.1 Data source

This study was conducted in Lanzhou, China, covering three counties and five districts. To ensure a representative sample, a stratified random cluster sampling strategy was employed. Schools were first stratified into primary and secondary levels, after which 28 public schools were randomly selected using a computer-generated random number sequence. All active, in-service teachers within these schools constituted the target population.

**Figure 1:**
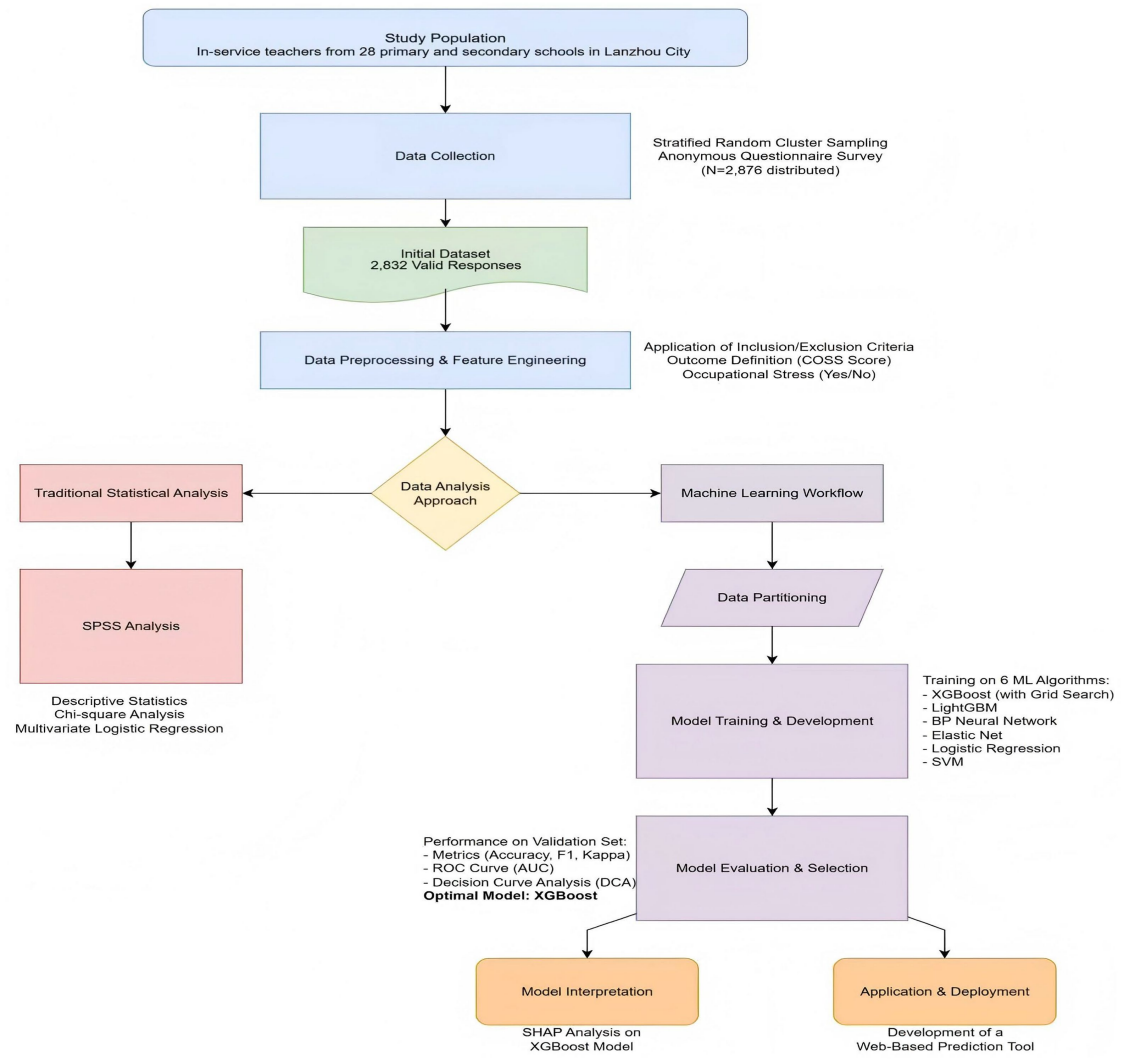
Flowchart of the study design and analysis pipeline.

A total of 2,876 questionnaires were administered to eligible teachers, from which 2,832 were returned complete and valid, resulting in a high effective response rate of 98.47%. Inclusion criteria were: (1) age ≥18 years, (2) continuous employment in the current role for at least 6 months. Exclusion criteria were: (1) self-reported family history of psychiatric disorders, (2) current use of psychotropic medications, (3) being on extended sick leave or having separated from employment (Figure 1).

Written informed consent was obtained from all adult participants prior to enrollment, emphasizing anonymity and the voluntary nature of the study. The detailed participant selection process is illustrated in Figure 1.

### 2.2 Data preprocessing

General Characteristics. Participants’ sociodemographic and occupational information was collected using a self-designed questionnaire informed by prior research (11, 12). The survey, which ensured participant anonymity, was conducted from October 2023 to May 2024. Key variables included age, sex, educational attainment, monthly income, marital status, weekly working hours, and two role-specific indicators: whether the teacher taught a graduating class and whether they held the position of a head teacher.

### 2.3 Screening predictors

The Core Occupational Stress Scale (COSS) was selected for this study primarily because it was specifically developed and validated for the Chinese occupational population. Compared to traditional western-derived tools, the COSS integrates key cultural and systemic factors relevant to the Chinese work environment across four dimensions: Social Support, Organization and Return, Demand and Effort, and Autonomy. Furthermore, its concise 17-item structure reduces respondent burden while maintaining high reliability (Cronbach’s α = 0.779), making it an ideal instrument for large-scale screening.

Following the classification criteria from a previous study, participants were categorized based on their total COSS score. Individuals with a score below 46 were classified as having “no stress,” while those with higher scores were further stratified into three severity levels: mild, moderate, and severe.

### 2.4 Baseline analysis

Statistical analyses were performed using IBM SPSS Statistics (Version 27.0). Continuous variables were expressed as mean ± standard deviation (SD) and compared using independent *t*-tests or ANOVA, while categorical data were analyzed using chi-square tests. For all tests, a *p*-value < 0.05 was considered statistically significant. Missing values were handled using listwise deletion given the high response rate (98.47%).

Machine learning (ML) procedures were implemented in R (Version 4.4.3) using the caret package. Continuous features (age and COSS scores) underwent *Z*-score normalization to ensure convergence for distance-based and gradient-descent algorithms (SVM, BP Neural Network, and Logistic Regression). Tree-based models (XGBoost and LightGBM) utilized raw data as they are invariant to feature scaling.

To identify independent predictors, a multivariate binary logistic regression model was constructed with occupational stress as the dichotomous outcome 0 = no, 1 = yes. This dichotomization facilitates risk stratification for public health interventions. Categorical variables were encoded using one-hot encoding prior to model training. Covariates were selected via a backward stepwise elimination procedure (*p* > 0.10 for removal).

### 2.5 Machine learning for variable screening

To develop the predictive models, the dataset was randomly partitioned into a training set (80%) and a hold-out validation set (20%), and the subsequent 5-fold cross-validation process.

All models were trained exclusively on the training data, and their final performance was evaluated on the unseen valid data to ensure an unbiased assessment of their generalization capabilities. For models requiring hyperparameter optimization, a systematic tuning process was conducted using cross-validation within the training set.

The study design and reporting followed the Transparent Reporting of a multivariable prediction model for Individual Prognosis Or Diagnosis (TRIPOD-AI) guidelines (13).

### 2.6 Model construction

To identify the optimal predictive model, we constructed and evaluated six distinct machine learning algorithms.

To ensure a rigorous and equitable comparison across diverse machine learning architectures, a standardized hyperparameter optimization pipeline was implemented using the caret framework within a 5-fold cross-validation scheme. Prior to model training, all numerical features underwent centering and scaling to eliminate dimensional disparities, ensuring an optimal input environment for algorithms sensitive to feature magnitude.

XGBoost: A comprehensive grid search was conducted to identify the optimal configuration. The search space included key parameters such as learning rate (eta: 0.01, 0.05, 0.1), maximum tree depth (max_depth: 3, 5, 7), row sampling proportion (subsample: 0.6, 0.8), column sampling proportion (colsample_bytree: 0.6, 0.8), and L2 regularization term (lambda: 0.1, 1). The optimal number of boosting iterations (nrounds) was determined automatically by the cross-validation process, which employed an early stopping criterion of 20 rounds to prevent overfitting (14).

Elastic Net Regression: This model was optimized using the cv.glmnet function. An alpha value of 1 was used, which configures the model as a Lasso regression, thereby enabling both regularization and feature selection. The function internally performs cross-validation to automatically select the optimal regularization strength (lambda), specifically the lambda.min value that minimizes cross-validated error (15).

LightGBM: The LightGBM model was optimized via a systematic grid search within the cross-validation framework. We tuned the learning rate and the number of leaves, with the optimal boosting iterations determined by the best-performing round to ensure maximum predictive accuracy (16).

Logistic regression: A standard logistic regression model was fitted using the glm function with a binomial family. As a parametric model, it does not have hyperparameters that require tuning in the same manner as the other machine learning models (17).

Support Vector Machine (SVM): Moving beyond default configurations, we performed a systematic grid search to optimize both the cost parameter (C) and the kernel parameter (sigma), ensuring the identification of the optimal separating hyperplane (18).

BP Neural Network: The neural network architecture was optimized by searching for the ideal number of hidden neurons (size) and the weight decay parameter. This grid-search approach enabled the model to balance complexity and generalization, rather than relying on pre-defined or default settings (19).

Model discrimination, the ability to correctly distinguish between participants with and without Occupational Stress, was primarily assessed by the Area Under the Receiver Operating Characteristic curve (AUC) (20). To provide a comprehensive assessment of classification performance, we also calculated several standard metrics from the confusion matrix: accuracy, precision, recall (sensitivity), specificity, F1-score, and Cohen’s Kappa coefficient (21, 22).

Accuracy, which measures the overall proportion of correct predictions among the total sample, is defined by Equation (1).


$$\text{Accuracy}=\frac{\mathrm{TP}+\mathrm{TN}}{\mathrm{TP}+\mathrm{TN}+\mathrm{FP}+\mathrm{FN}}$$ (1)

Precision, which quantifies the proportion of positive predictions that were genuinely correct, is calculated using Equation (2)


$$\text{Precision}=\frac{\mathrm{TP}}{\mathrm{TP}+\mathrm{FP}}$$ (2)

Recall (also known as sensitivity), which reflects the model’s ability to correctly identify all true positive cases, is presented in Equation (3).


$$\text{Recall}=\frac{\mathrm{TP}}{\mathrm{TP}+\mathrm{FN}}$$ (3)

The F1-score, representing a balanced measure between precision and recall through their harmonic mean, is calculated using Equation (4).


$$F1-\text{Score}=\frac{2\mathrm{TP}}{2\mathrm{TP}+\mathrm{FP}+\mathrm{FN}}$$ (4)

Specificity, which measures the proportion of actual negatives that were correctly identified by the model, is given by Equation (5).


$$\text{Specificity}=\frac{\mathrm{TN}}{\mathrm{TN}+\mathrm{FP}}$$ (5)

The Cohen’s Kappa coefficient was used to evaluate the agreement beyond chance. Model calibration, which measures the agreement between predicted probabilities and observed outcomes, was visually assessed using calibration plots and quantified with the Hosmer-Lemeshow goodness-of-fit test (13, 23).

### 2.7 Model evaluation

We evaluated the final model’s performance across three primary dimensions: its discriminative power, clinical utility, and generalizability. We utilized the Area Under the Receiver Operating Characteristic curve (AUROC) to measure discrimination, while Decision Curve Analysis (DCA) assessed the model’s net benefit. DCA was employed to assess and compare the net benefit of using each model for clinical decision-making across a range of clinically relevant threshold probabilities (23).

## 3 Results

### 3.1 Demographic characteristics

A total of 2,832 teachers were included in the final analysis. The demographic characteristics of the participants are detailed in Table 1. Of the participants, 748 (26.4%) were male and 2,084 (73.6%) were female. Regarding the school level, 1,350 teachers (47.7%) were from primary schools, and 1,482 (52.3%) were from secondary schools.

**Table 1:** Analysis of occupational stress prevalence across different teacher characteristics.

Characteristic	Group	*N*	Occupational stress detection rate (%)				*χ*^2^ value	*p*-value
			None	Mild	Moderate	Severe		
School type	Primary School	1,350	68.6	18.8	7.6	5	6.527	0.089
	Secondary School	1,482	65	19.3	9.2	6.5		
Sex	Male	748	61.5	21.4	10.7	6.4	13.82	**0.003**
	Female	2084	68.6	18.2	7.6	5.6		
Age	20–35 years	953	72	16.1	7.8	4.2	21.949	**0.001**
	36–45 years	966	63.6	20.1	9.1	7.2		
	≥46 years	913	64.5	21.1	8.4	5.9		
Education level	Junior college and below	228	67.5	19.7	9.6	3.1	6.945	0.326
	Bachelor’s degree	2,408	66.2	19.3	8.5	6		
	Postgraduate and above	196	71.9	15.8	6.1	6.1		
Monthly income	<5,000 RMB	1,398	65.5	19.8	8.4	6.3	2.608	0.456
	>5,000 RMB	1,434	67.9	18.3	8.5	5.3		
Marital status	Married	2,368	65.7	19.9	8.6	5.8	13.279	**0.039**
	Unmarried	392	73.7	13.8	7.7	4.8		
	Divorced or widowed	72	62.5	20.8	6.9	9.7		
Years of service	6–10 years	952	71.3	16.6	7.5	4.6	16.137	0.185
	11–15 years	361	64	20.2	8.9	6.9		
	16–20 years	325	64.6	18.5	9.8	7.1		
	20–25 years	484	64.5	20.7	8.9	6		
	>25 years	710	64.4	21	8.6	6.1		
Weekly working hours	≤40 h	989	75.6	14.2	7.8	2.4	122.717	**<0.001**
	41–48 h	1,127	67.3	20.3	7.2	5.2		
	49–56 h	445	54.4	23.4	11.9	10.3		
	>56 h	271	52	24.7	10.3	12.9		
Household registration	Local	2,406	66	19.5	8.2	6.3	10.479	**0.015**
	Non-local	426	70.9	16.4	9.6	3.1		
Teaching a graduating class	Yes	880	63.5	19.8	10.1	6.6	7.919	**0.048**
	No	1952	68.1	18.8	7.7	5.4		
Head teacher	Yes	942	65.7	21.2	6.8	6.3	8.744	**0.033**
	No	1890	67.2	18	9.3	5.6		
Exercise frequency	Daily	363	72.7	16.5	8	2.8	103.62	**<0.001**
	1–3 times/month	960	68.6	19.3	7.5	4.6		
	1–3 times/week	594	73.4	16.3	6.9	3.4		
	4–6 times/week	129	76.7	14.7	7	1.6		
	Never	786	54.8	22.8	11.2	11.2		
Hobbies	Literature	484	66.1	19.6	6.8	7.4	33.532	**<0.001**
	Music	663	69.4	16.1	9.7	4.8		
	Sports	451	67.6	20	9.8	2.7		
	Film/TV	594	69.5	18.5	6.1	5.9		
	None/Other	640	61.1	21.6	9.7	7.7		

*N*, number of participants; *χ*^2^, Chi-square value, representing the test statistic for group differences. *p*-value: Statistical significance (bold indicates *p* < 0.05). Occupational Stress: Defined by the Core Occupational Stress Scale (COSS). Detection Rate: Calculated as the percentage of participants within each group identified with mild, moderate, or severe stress. RMB, Renminbi (Chinese currency).

The mean total score on the Core Occupational Stress Scale (COSS) for all participants was 46.02 ± 9.63. The mean scores for the four sub-dimensions were as follows: Social Support (16.12 ± 5.15), Organization and Return (13.41 ± 3.55), Demand and Effort (10.06 ± 3.72), and Autonomy (16.43 ± 1.88). As shown in Table 1, statistically significant differences in the total COSS scores were observed across various subgroups, including school type, sex, age, marital status, work tenure, weekly working hours, teaching a graduating class, exercise frequency, and hobbies in Table 1 (all *p* < 0.05).

### 3.2 Feature selection

The overall prevalence of occupational stress in the study cohort was 33.3%, with 943 out of 2,832 teachers meeting the criteria. Of those experiencing stress, 19.1% were categorized as mild, 8.4% as moderate, and 5.8% as severe. A chi-square analysis, with full results presented in Table 1, was conducted to explore the relationship between various factors and the presence of occupational stress. This unadjusted analysis revealed that the prevalence of stress varied significantly by sex, age, marital status, household registration, weekly working hours, teaching a graduating class, role as a head teacher, frequency of exercise, and type of hobby in Table 2 (all *p* < 0.05).

**Table 2:** Multivariate logistic regression analysis of factors associated with occupational stress.

Characteristic	Group	*N*	*B*	S.E.	Wald χ^2^	*p*-value	OR	95% CI
Sex	Male	748	0.266	0.102	6.776	0.009	1.305	1.068–1.594
	Female	2084					1	
Age	≥46 years	953	0.284	0.126	5.031	0.025	1.328	1.036–1.702
	36–45 years	966	0.309	0.12	6.617	0.01	1.362	1.076–1.723
	20–35 years	913					1	
Weekly working hours	>57 h	989	1.051	0.142	54.532	<0.001	2.862	2.165–3.783
	41–48 h	1,127	0.413	0.098	17.901	<0.001	1.511	1.248–1.829
	49–56 h	445	0.957	0.121	62.952	<0.001	2.604	2.055–3.298
	≤40 h	271					1	
Exercise frequency	Daily	363	−0.787	0.138	32.541	<0.001	0.455	0.347–0.597
	1–3 times/month	960	−0.59	0.1	34.846	<0.001	0.555	0.456–0.674
	1–3 times/week	594	−0.821	0.117	48.993	<0.001	0.44	0.35–0.554
	4–6 times/week	129	−1	0.22	20.585	<0.001	0.368	0.239–0.567
	Never	786					1	
Hobbies	Literature	484	−0.211	0.132	2.545	0.111	0.81	0.624–1.049
	Music	663	−0.315	0.121	6.801	0.009	0.73	0.576–0.925
	Sports	451	−0.381	0.138	7.607	0.006	0.683	0.521–0.896
	Film/TV	594	−0.289	0.126	5.248	0.022	0.749	0.585–0.959
	Other/None	640					1	

B: Unstandardized regression coefficient; a positive value indicates a risk factor, while a negative value indicates a protective factor. S.E, standard error, reflecting the sampling variability of the coefficient. Wald *χ*^2^, Wald chi-square statistic, used to test the unique contribution of each predictor. *p*-value: Statistical significance level (typically considered significant if *p* < 0.05). OR (Odds Ratio): Represents the odds of developing occupational stress given a specific exposure compared to the reference group. 95% CI, 95% confidence interval for the odds ratio; if the interval does not include 1.0, the result is statistically significant.

### 3.3 Model performance and validation

In the validation set analysis, the XGBoost model consistently demonstrated higher clinical net benefit than Logistic Regression, as evidenced by the DCA curves. This justifies the use of ensemble learning to capture complex non-linear interactions between sociodemographic and lifestyle factors that traditional linear models may overlook.

The LightGBM and BP Neural Network models showed clear evidence of severe overfitting. Although they attained high accuracy on the training set (0.745 and 0.744, respectively), their performance degraded significantly on the validation set, with accuracies dropping to 0.572 and 0.523. This substantial performance gap suggests that these models learned idiosyncratic noise from the training data rather than generalizable predictive patterns, rendering them unreliable for practical use.

The Logistic Regression and Elastic Net models provided stable but moderate performance, serving as effective benchmarks. The Support Vector Machine (SVM) model, however, was unsuitable for this dataset, as indicated by its negative Kappa coefficient on both the training (−0.297) and validation (−0.091) sets, meaning its predictive power was worse than random chance.

Given its superior predictive accuracy on unseen data, robust generalization capabilities, and overall balanced performance, the XGBoost model was selected as the optimal model for predicting occupational stress in this study. Subsequent analyses, including Receiver Operating Characteristic (ROC) curves and Decision Curve Analysis (DCA), will further elaborate on the comparative performance of these models.

**Figure 2:**
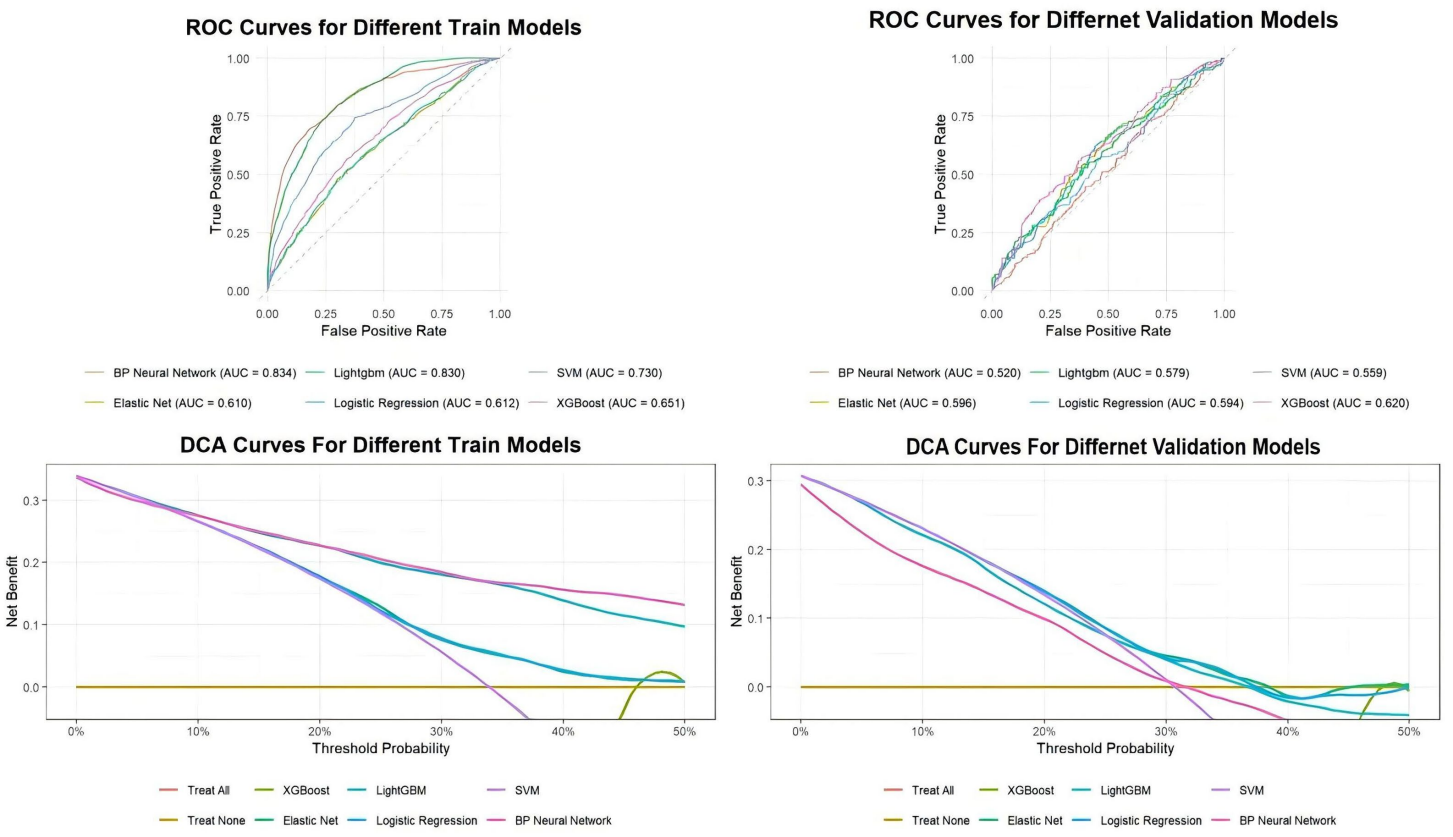
ROC and DCA curves for all models in the validation and training set.

To visually assess and compare the discriminative ability and clinical utility of the six predictive models, Receiver Operating Characteristic (ROC) curves and Decision Curve Analysis (DCA) were generated for both the training and validation set (Figure 2).

The ROC curves, which plot the true positive rate against the false positive rate, illustrate the models’ ability to distinguish between teachers with and without occupational stress. On the training set, the BP Neural Network (AUC = 0.834) and LightGBM (AUC = 0.830) models exhibited excellent discrimination. However, this high performance was indicative of overfitting, as their discriminative power substantially decreased on the validation set.

The validation set results are critical for evaluating a model’s generalizability. On the validation set, the XGBoost model demonstrated the best discriminative performance, achieving the highest Area Under the Curve (AUC) of 0.620. This was closely followed by the Logistic Regression (AUC = 0.594) and Elastic Net (AUC = 0.596) models. The performance of the BP Neural Network (AUC = 0.520) and SVM (AUC = 0.559) was notably weaker, approaching the performance of a random classifier (AUC = 0.5).

Decision Curve Analysis (DCA) was employed to evaluate the clinical net benefit of each model across a range of threshold probabilities. A model is considered to have clinical value if its curve is above the “Treat All” and “Treat None” strategies.

On the training set, most models showed a positive net benefit. However, the DCA results on the validation set (Figure 2) provide a more realistic assessment of clinical utility. In the validation set analysis, the XGBoost model (green curve) again demonstrated superior performance, providing the highest net benefit across a clinically relevant range of threshold probabilities, particularly between approximately 35% and 50%. While other models like Logistic Regression and Elastic Net also offered a positive net benefit, their curves were consistently lower than that of the XGBoost model. The SVM and BP Neural Network models showed limited to no clinical utility, with their curves often falling below the “Treat All” baseline.

Both the ROC and DCA analyses consistently support the selection of the XGBoost model as the optimal predictive tool in Table 3. It not only possesses the strongest ability to discriminate between high- and low-risk individuals but also offers the greatest potential for clinical net benefit when applied to new, unseen populations.

**Table 3:** Performance metrics of six machine learning models on the training and validation sets.

Dataset	Metric	XGBoost	Elastic net	LightGBM	Logistic regression	SVM	BP neural network
Validation set (*N* = 567)	Accuracy	0.603	0.558	0.572	0.578	0.447	0.523
	Precision	0.765	0.773	0.745	0.768	0.645	0.709
	Recall	0.615	0.513	0.582	0.559	0.449	0.528
	F1 Score	0.682	0.617	0.653	0.647	0.443	0.605
	SPE	0.575	0.661	0.552	0.621	0.443	0.512
	NPV	0.398	0.376	0.369	0.384	0.263	0.325
	Kappa	0.169	0.143	0.117	0.153	−0.091	0.034
	TP	100	115	96	108	77	89
	TN	241	201	228	219	176	207
	FP	74	59	78	66	97	85
	FN	151	191	164	173	216	185
Training set (*N* = 2,265)	Accuracy	0.628	0.572	0.745	0.591	0.307	0.744
	Precision	0.749	0.73	0.856	0.732	0.458	0.857
	Recall	0.659	0.559	0.738	0.601	0.272	0.735
	F1 Score	0.701	0.633	0.792	0.66	0.341	0.791
	SPE	0.57	0.598	0.758	0.572	0.375	0.761
	NPV	0.462	0.411	0.597	0.424	0.209	0.596
	Kappa	0.216	0.142	0.465	0.16	−0.297	0.464
	TP	438	460	583	440	288	585
	TN	986	837	1,104	899	407	1,100
	FP	331	309	186	329	481	184
	FN	511	660	393	598	1,090	397

XGBoost, extreme gradient boosting; LightGBM, light gradient boosting machine; SVM, support vector machine; BP, backpropagation (neural network); LR, logistic regression; EN, elastic net; AUC, area under the receiver operating characteristic curve. Accuracy: (TP + TN) / (TP + TN + FP + FN); Precision: TP / (TP + FP); Recall (Sensitivity): TP / (TP + FN); F1 Score: Harmonic mean of Precision and Recall; SPE (Specificity): TN / (TN + FP); NPV (Negative Predictive Value): TN / (TN + FN); Kappa: Cohen’s kappa coefficient, used to measure the agreement between predicted and actual results; TP, true positive; TN, true negative; FP, false positive; FN, false negative.

The predictive performance of the six machine learning models was comprehensively evaluated on both the training and the hold-out validation set. A detailed summary of all performance metrics, including accuracy, precision, recall, F1 score, specificity (SPE), and Cohen’s Kappa, is presented in Table 3.

The primary measure of a model’s real-world utility is its performance on the validation set, which reflects its ability to generalize to new data. In this crucial evaluation, the XGBoost model demonstrated superior and the most well-balanced performance. It achieved the highest Accuracy (0.603), F1 Score (0.682), and Cohen’s Kappa coefficient (0.169) on the validation set. Importantly, the performance metrics between the training set (Accuracy: 0.628) and the validation set (Accuracy: 0.603) were closely aligned, indicating that the XGBoost model is robust and does not suffer from significant overfitting.

### 3.4 Outcome of SHAP value

To enhance the interpretability of the optimal XGBoost model and to understand the underlying drivers of its predictions, a SHAP (SHapley Additive exPlanations) analysis was conducted. The results, visualized in Figures 3A,B, provide insights into both the global feature importance and the local impact of each feature on individual predictions.

**Figure 3A:**
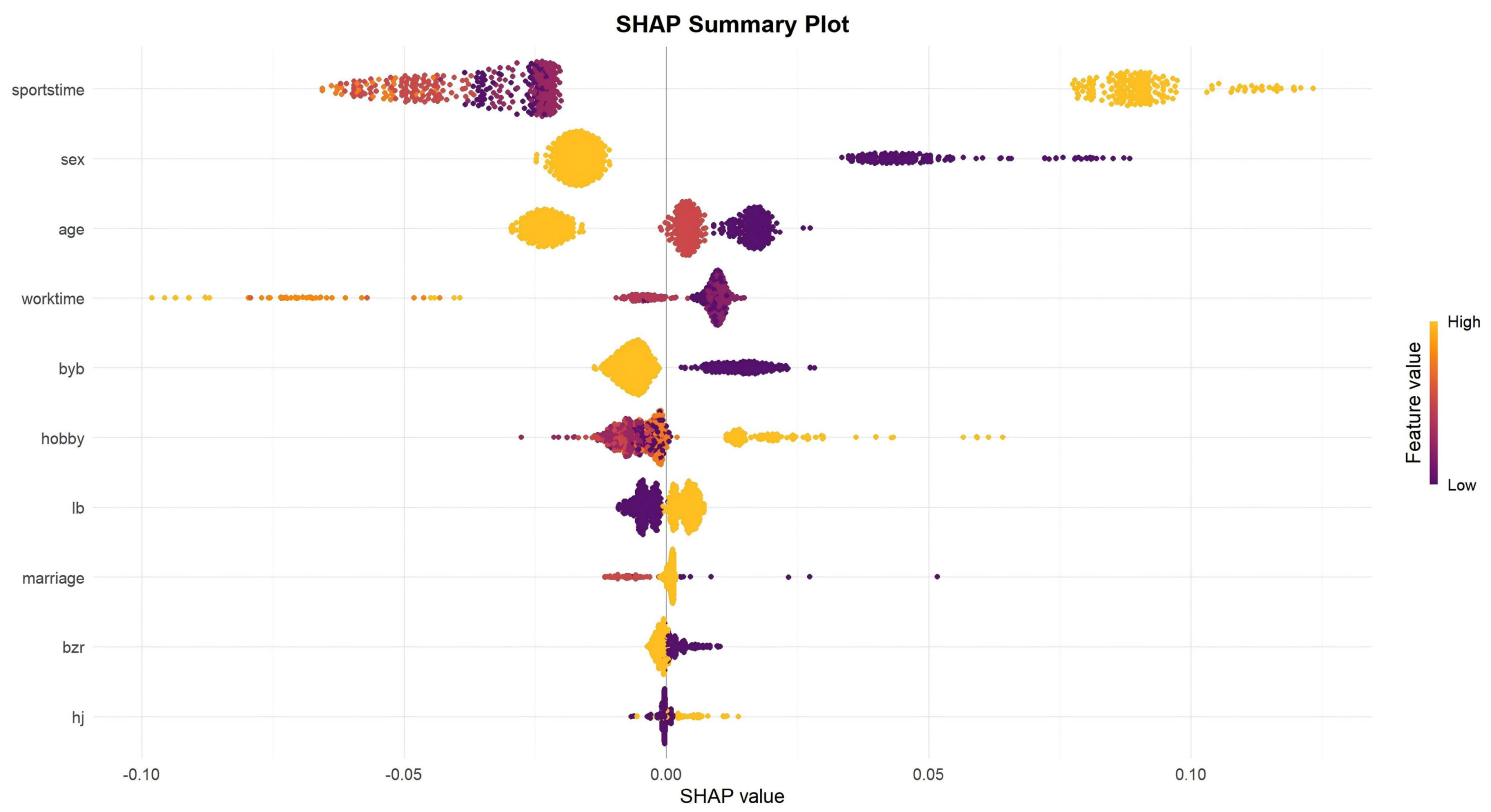
The SHAP summary plot. This plot visualizes the direction and magnitude of each feature’s impact, with points colored by feature value from low (purple) to high (yellow).

The SHAP feature importance plot (Figure 3B) ranks the features based on their mean absolute SHAP value, which quantifies the average magnitude of their impact on the model’s output. The analysis reveals that weekly exercise time (sportstime) was by far the most influential predictor of occupational stress. This was followed by sex and age, which also demonstrated substantial predictive power. Other significant contributors included weekly working hours (worktime), teaching a graduating class, and having a hobby. In contrast, features such as teacher category, marital status (marriage), serving as a head teacher, and household registration had a relatively minor impact on the model’s predictions.

**Figure 3B:**
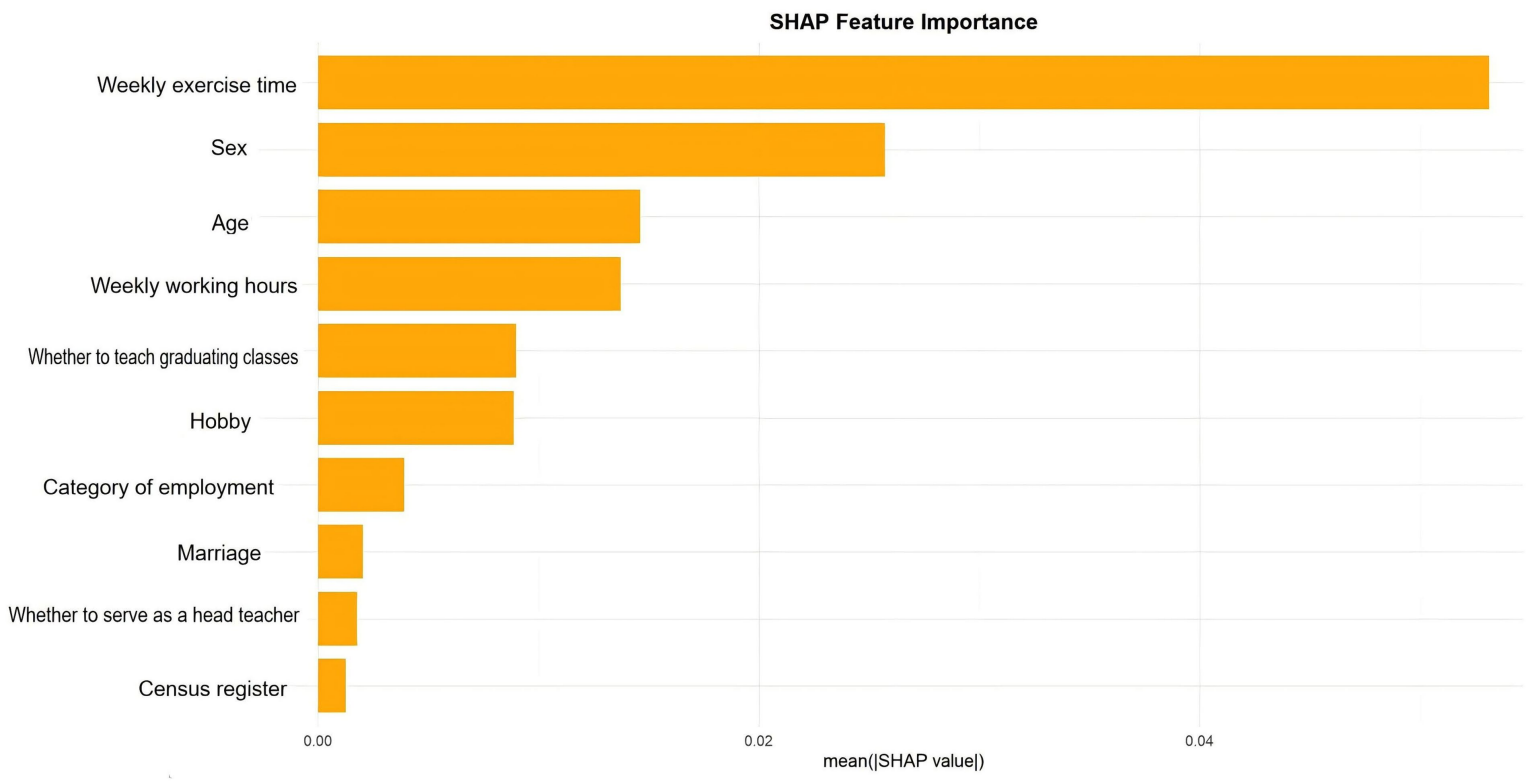
The SHAP Feature Importance horizontal bar chart. This chart ranks ten features by their mean absolute SHAP value.

The SHAP summary plot (Figure 3A) offers a more nuanced view by illustrating not only the magnitude but also the direction of each feature’s effect. Each point on the plot represents a single teacher in the validation set.

Weekly Exercise Time is a clear trend was observed where lower feature values (representing more frequent exercise) are predominantly associated with negative SHAP values. This strongly suggests that engaging in more frequent exercise is a protective factor, significantly lowering the predicted risk of occupational stress. Conversely, the highest feature value (never)is linked to positive SHAP values, increasing the risk.

The plot shows a distinct separation based on sex. Higher feature values (female) are overwhelmingly associated with negative SHAP values, indicating a lower predicted risk of occupational stress. Conversely, lower feature values (male) are consistently linked to positive SHAP values, confirming that being male is a significant risk factor according to the model.

For age, lower feature values (older age groups) tend to have positive SHAP values, suggesting that advancing age is a risk factor for occupational stress. Higher feature values (20–35 = 3) are associated with negative SHAP values, indicating lower risk.

Weekly Working Hours is a clear pattern emerges where lower feature values (representing longer working hours) consistently push the prediction towards a higher risk of occupational stress (positive SHAP values). Higher feature values (≤40 h) are associated with negative SHAP values, acting as a protective factor.

These SHAP results provide transparent, quantitative evidence of how the XGBoost model synthesizes individual teacher characteristics to arrive at a personalized risk prediction, confirming the critical roles of lifestyle factors, demographic characteristics, and work-related demands in assessing occupational stress.

### 3.5 Development of a web-based prediction tool

To facilitate the practical application and accessibility of our findings, the final, validated XGBoost model was deployed as an interactive, user-friendly online prediction tool. This web-based calculator was designed to provide teachers and educational administrators with an immediate, individualized risk assessment for occupational stress based on the key predictors identified in this study.

**Figure 4:**
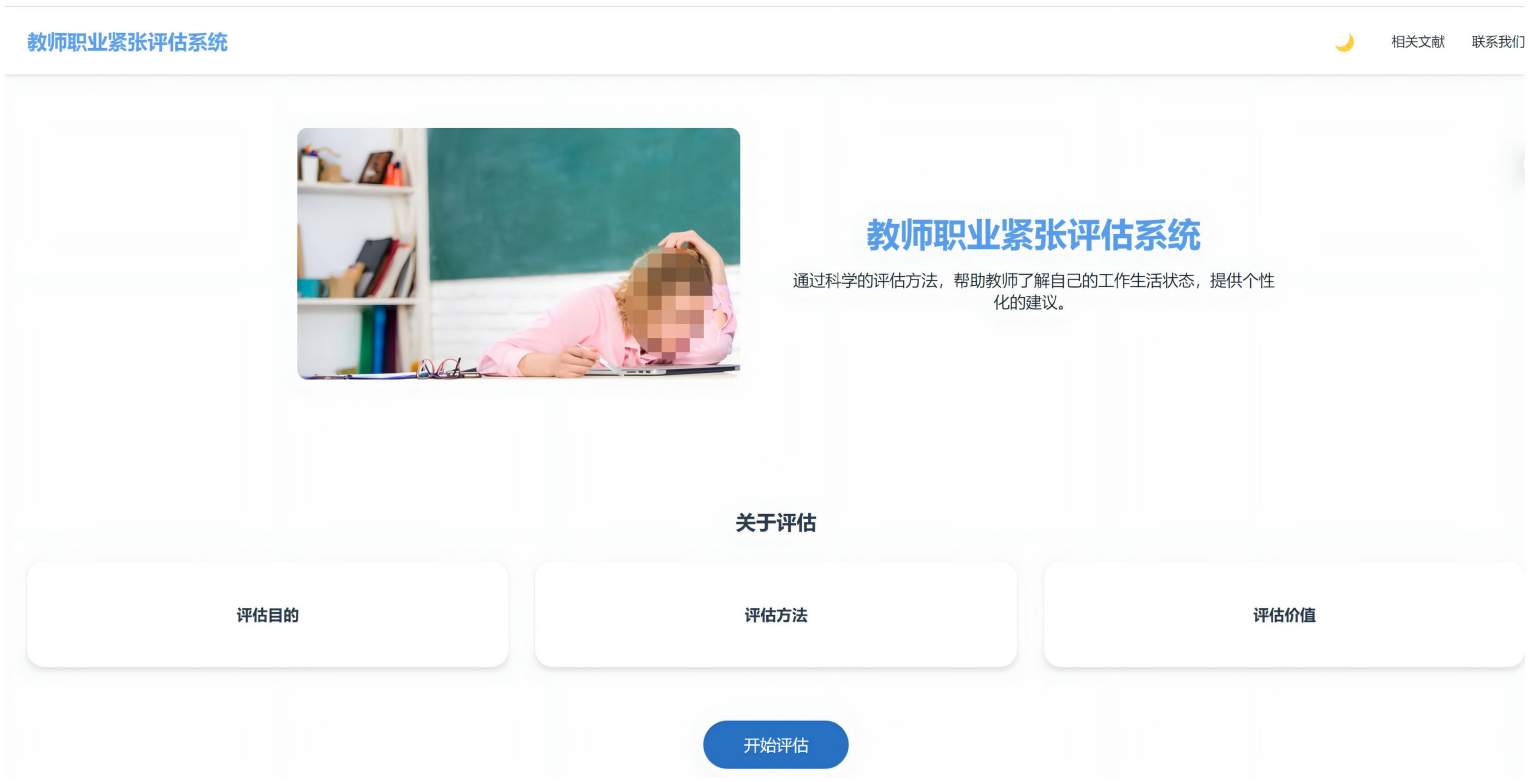
Interface of the web-based predictive tool for teacher occupational stress (https://teacher-evaluation-olive.vercel.app/).

The interface of the web tool, as shown in Figure 4 prompts the user to input specific values for the model’s predictor variables. These include demographic information (sex, age), work-related factors (weekly working hours, whether teaching a graduating class, role as a head teacher), and lifestyle habits (weekly exercise frequency, hobbies). All input variables are easily self-reported and do not require any invasive clinical measurements.

Upon submission of the data, the underlying XGBoost algorithm processes the inputs and instantly calculates a personalized probability score, representing the individual’s predicted risk of experiencing occupational stress. This output is presented in a clear and understandable format.

The primary objective of this tool is to bridge the gap between complex data-driven research and real-world application. It empowers individual teachers with a means for proactive self-assessment and encourages early awareness of potential risks. For educational institutions and health policymakers, it can serve as a rapid, cost-effective screening instrument to identify high-risk groups, enabling the targeted allocation of support resources and the implementation of preventative interventions. The web-based predictor is publicly accessible at: [https://teacher-evaluation-olive.vercel.app/].

## 4 Discussion

This study developed and validated a machine learning framework for predicting occupational stress among teachers, with XGBoost emerging as the superior model (AUC = 0.620). While an AUC of 0.620 represents modest discrimination, its value should be interpreted within the context of public health screening rather than clinical diagnosis. As demonstrated by our DCA curves, the XGBoost model offers significant clinical net benefit, enabling effective risk stratification. This allows educational institutions to proactively identify high-risk teacher subgroups for early intervention, optimizing the allocation of limited mental health resources (24–26).

By leveraging the SHAP (SHapley Additive exPlanations) method for model interpretation, we identified weekly exercise time, sex, and age as the most influential predictors (27).

Consistent with a large body of evidence linking extended work hours to adverse psychological outcomes, our model identified long working hours as a major risk factor. Specifically, we quantified this risk, showing that teachers working over 41 h per week faced a progressively increasing risk of stress, with those exceeding 57 h being the most vulnerable. This echoes international concerns regarding teacher workload and its impact on well-being (27).

Notably, our model identified male teachers as having a higher risk of occupational stress, which contrasts with some previous findings regarding female emotional labor (28). This disparity may be driven by gender-specific coping strategies or male teachers’ potential reluctance to seek social support amidst high organizational demands (29, 30).

In addition to these risk factors, the strong protective effect of regular physical exercise is a well-established principle in public health, and our study robustly confirms its importance in the context of teacher stress. The dose–response relationship identified by the model—where more frequent exercise corresponded to a lower risk—provides strong evidence for promoting physical activity as a primary intervention strategy (31, 32).

This study possesses several key strengths. To our knowledge, it is one of the first to apply a rigorous machine learning framework to predict occupational stress among Chinese teachers. By utilizing XGBoost, we successfully captured complex, non-linear relationships that traditional linear models often overlook. Furthermore, the integration of SHAP methodology addresses the “black box” nature of machine learning, providing transparent, individualized explanations that are crucial for clinical trust. The most significant practical contribution is the development of a user-friendly, web-based prediction tool. This translates academic findings into a tangible instrument, empowering teachers with a confidential means for self-assessment and bridging the gap between research and proactive health management.

Despite these contributions, several limitations warrant consideration. First, the cross-sectional design precludes causal inference; longitudinal studies are required to confirm the causal links between predictors and stress onset. Second, self-reported data may be subject to recall or social desirability bias. Third, while our sample is representative of Lanzhou, its generalizability to rural or different cultural contexts remains to be established through external validation.

Finally, our use of binary classification (stress vs. no stress) facilitates risk stratification but may lose nuanced information regarding severity. Future research should prioritize multi-class prediction models (e.g., mild, moderate, and severe) to allow for more granular, tailored intervention strategies.

In conclusion, our findings offer critical implications for educators and policymakers. The identification of high-risk subgroups enables targeted wellness initiatives and workload management. Ultimately, this research underscores the urgent need for systemic changes, including stricter regulations on working hours and the promotion of school-based mental health programs, to mitigate the prevalence of occupational stress in the teaching profession.

## 5 Conclusion

In conclusion, this study demonstrates that machine learning, specifically the XGBoost algorithm, provides a powerful and effective method for predicting occupational stress among primary and secondary school teachers using non-invasive, self-reported data. The validated model and its deployment as a public web tool offer a significant step forward from traditional assessment methods, providing a promising, data-driven approach for the early identification of at-risk teachers and enabling targeted interventions to safeguard their well-being.

## Data Availability

The datasets presented in this article are not readily available because the data that support the findings of this study are not publicly available due to strict ethical restrictions regarding patient confidentiality and privacy. Participants of this study did not consent for their data to be shared publicly. Requests to access the datasets should be directed to 575752799@qq.com.
